# Synergistic Anti-Tumor Effect of Simvastatin Combined to Chemotherapy in Osteosarcoma

**DOI:** 10.3390/cancers13225869

**Published:** 2021-11-22

**Authors:** Adèle Mangelinck, Nadia Habel, Audrey Mohr, Nathalie Gaspar, Bojana Stefanovska, Olivia Fromigué

**Affiliations:** 1 Inserm, UMR981, Gustave Roussy Cancer Campus, Paris-Saclay University, F-94805 Villejuif, France; adele.mangelinck@gmail.com (A.M.); nadia.habel@inrae.fr (N.H.); bojana_06@hotmail.com (B.S.); 2 Ecole Nationale Supérieure de Chimie de Montpellier, F-34090 Montpellier, France; 3 Inserm, U1015, Gustave Roussy Cancer Campus, Paris-Saclay University, F-94805 Villejuif, France; audrey.mohr@gustaveroussy.fr (A.M.); nathalie.gaspar@gustaveroussy.fr (N.G.); 4 Child and Adolescent Cancer Department, Gustave Roussy Cancer Campus, F-94805 Villejuif, France

**Keywords:** chemoresistance, adjuvant therapy, bone tumor, invasion, mevalonate pathway, statin

## Abstract

**Simple Summary:**

Osteosarcoma is the most common form of primary solid bone malignancy, with the highest incidence in adolescence. The therapeutic management includes surgical resection combined with adjuvant/neoadjuvant chemotherapy regimens. Despite this multimodal combination, about two patients out of five are still not cured (5-year overall survival rate at 60%). Complementary therapeutic approaches are required to overcome the frequent resistance to conventional chemotherapy. The aim of the present study was to assess the potential benefit of statins as an adjuvant to chemotherapy. We show that simvastatin synergizes with conventional chemotherapy drugs in terms of cell viability, tumor growth, and dissemination and represents valuable alternative adjuvant therapy that needs further investigation in clinical trials.

**Abstract:**

Context: Osteosarcoma is the most common primary solid malignancy of the bone, mainly affecting pediatric patients. The main clinical issues are chemoresistance and metastatic spread, leading to a survival rate stagnating around 60% for four decades. Purpose: Here, we investigated the effect of simvastatin as adjuvant therapy on chemotherapy. Methods: Cell viability was assessed by the MTT test, and a combination index was evaluated by an isobologram approach. Cell motility was assessed by wound-healing assay. Cell-derived xenograft models were established in mice. FFPE tumor samples were assessed by immunohistochemistry. Results: In vitro experiments indicate that simvastatin synergized the conventional chemotherapy drugs’ inhibitory effect on cell viability. Functional assays reveal that simvastatin supplementation favored the anticancer mechanism of action of the tested chemotherapy drugs, such as DNA damage through intercalation or direct alkylation and disorganization of microtubules. Additionally, we show that even though simvastatin alone did not modify tumor behavior, it potentiated the inhibitory effect of doxorubicin on primary tumor growth (+50%, *p* < 0.05) and metastatic spread (+50%, *p* < 0.05). Our results provide evidence that simvastatin exerted an anti-tumor effect combined with chemotherapy in the preclinical murine model and represents valuable alternative adjuvant therapy that needs further investigation in clinical trials.

## 1. Introduction

Osteosarcoma is the most common primary malignant tumor of the bone, mainly affecting children, teenagers, and young adults under the age of 20. Adjuvant and neoadjuvant systemic chemotherapy regimens are considered the standard treatment for the past 40 years as they significantly improve the survival of patients [1]. The histological response to treatment represents a reliable clinical indicator factor in predicting the overall survival since poor responders to chemotherapy usually have the worse prognosis. Despite numerous studies, the key mechanism of chemoresistance in patients with osteosarcoma is still inconclusive. That points to the need for additional adjuvant therapeutic strategies.

Statins are widely used in clinical practice in the context of hypercholesterolemia-derived cardiovascular and coronary heart diseases [2]. These compounds indeed inhibit the activity of the 3-hydroxy-3-methylglutaryl-coenzyme-A reductase (HMGCR) enzyme, the major rate-limiting enzyme in cholesterol neosynthesis [3]. By preventing the conversion of HMG-coenzyme A into mevalonic acid, statins also prevent the production of downstream metabolites such as the isoprenoid residues geranylgeranyl pyrophosphate and farnesyl pyrophosphate. In addition to the inhibition of cholesterol neosynthesis, those isoprenoid residues exert pleiotropic effects on several essential cellular functions, including cell proliferation, differentiation, and survival, but also the regulation of cell morphology and motility.

Several epidemiological studies have provided evidence of reduced cancer incidence and mortality with the use of statins [4,5,6]. This resulted in great interest in the therapeutic potential of statins in malignant tumors. In preclinical studies, combinations of statins with anticancer drugs or cytotoxic chemotherapy exhibit synergistic effects on various cancer cell types [7]. Clinical trials investigating combination therapy with statins have displayed encouraging results with better outcomes compared to monotherapy [6,8,9].

We and others have previously shown that statins exert an anti-tumor effect in vitro, induce programmed cell death, decrease cell migration and invasion capacities, and strengthen the cytotoxic effect in combination with standard drugs on osteosarcoma cells [10,11,12,13]. However, the anti-tumor potential of statins in animal models of osteosarcoma remains largely unexplored. In the present study, we highlight the synergistic effect of simvastatin in combination with commonly used chemotherapeutic drugs compared to monotherapy in a murine model of osteosarcoma, validating the previous promising in vitro data in preclinical conditions.

## 2. Materials and Methods

### 2.1. Reagents

Etoposide (CAS number 33419-42-0) was purchased as a 20 mg·mL^−1^ injectable solution from Teva Santé (Paris, France). Methotrexate (CAS number RN 59-05-2) was purchased as a 55 mM injectable solution from Mylan (Saint Priest, France). All other compounds (Table 1) were purchased as a powder from Sigma-Aldrich (Lyon, France).

### 2.2. Cell Culture

Murine osteosarcoma cell line K7M2 was purchased from the American Type Culture Collection (ATCC; LGC Standards Sarl; Molsheim, France) and amplified to generate a cell master bank. All experiments were carried out from this cell bank.

Cells were cultured in high glucose Dulbecco’s modified Eagle medium (DMEM, Invitrogen, Saint Aubin, France) supplemented with 10% heat-inactivated fetal calf serum, at 37 °C in a humidified atmosphere (5% CO_2_ and 95% air). Culture media were changed three times a week and regularly tested for mycoplasma contamination (PCR-based assay from Minerva-Biolabs, Berlin, Germany).

### 2.3. Plasma Membrane Permeability Assay

The cell membrane permeability assay was adapted from [14]. Cells were seeded at a density of 30,000 cells·cm^−2^, labeled with calcein-AM (5 µM; Sigma-Aldrich) for 2 h and washed with PBS to remove residual calcein-AM. Cells were then incubated with indicated concentrations of simvastatin for 16 h. Plasmolysis was induced by injection of a hyperosmotic solution (400 mM sucrose), and fluorescence release kinetics was quantified using a luminescence microplate reader.

### 2.4. Cell Metabolic Activity

Cells were seeded at a density of 12,000 cells·cm^−2^ and allowed to adhere for 24 h before treatment with the indicated compounds. Cell metabolic activity was evaluated by incubation with MTT reagent (3-(4,5-dimethylthiazol-2-yl)-2,5-diphenyltetrazolium bromide) at 0.1 mg/mL for 2 h. The medium was discarded, the intracellular insoluble formazan was solubilized in dimethyl sulfoxide (DMSO), and the absorbance was read spectrophotometrically at 592 nm.

Isoboles (i.e., isoeffective-curves) were established from experimental data for drugs used alone and in different fixed concentration ratios. These data are plotted on an isoeffective graph with axes representing the doses of each drug. The level of synergism between drugs was evaluated according to the quantitative measure originally described by [15]. This method is based on the Loewe additivity model transcribed by the equation [D1]/[Dx1] + [D2]/[Dx2] = 1 where [Di] and [Dxi] are the respective half-maximal inhibitory concentration (IC_50_) of drug i when acting alone and in combination preparation. The combination index (CI) derives from the same equation, CI = D1/Dx1 + D2/Dx2. A CI of less than, equal to, and more than 1 classifies drug interactions as synergistic, additive (zero interaction), or antagonistic, respectively.

### 2.5. Subcellular Fractionation

The cytosolic and membrane fractions were isolated as described [16]. Briefly, cells were sonicated in 10 mM Tris/HCl (pH 7.4) before the addition of Triton-X114 (1%). Samples were vortexed for 2 min then incubated at 31 °C for 4 min. Lysates were centrifuged at 300× *g* for 3 min at RT. The upper aqueous phase was collected and submitted to a second round of extraction with Triton X-114. Both detergent phases that contained the membrane fractions were pooled. The aqueous phase corresponded to the cytosolic fraction. Protein concentration in the aqueous phase was determined using the DC protein assay (Bio-Rad Laboratories, Marnes-la-Coquette, France).

### 2.6. Western Blot Analysis

Proteins (30 µg) were resolved by sodium dodecyl sulfate-polyacrylamide gel electrophoresis (SDS-PAGE) and transferred onto nitrocellulose membranes. Membranes were saturated for 2 h at RT in casein blocking buffer (Sigma-Aldrich), then incubated overnight at +4 °C with specific primary antibodies (1/1000 in blocking buffer). Mouse anti-RhoA was purchased from Santa Cruz Biotechnology (Heidelberg, Germany), and rabbit anti-Actin was purchased from Sigma-Aldrich. Membranes were washed three times with TBST buffer (50 mM Tris/HCl (pH 7.4), 150 mM NaCl, 0.1% (*v*/*v*) Tween-20), and incubated for 2 h at RT with appropriate horseradish peroxidase (HRP)-conjugated secondary antibody (1/20,000 in blocking buffer). After final washes, the signals were visualized with the enhanced chemiluminescence Western blotting detection reagent (ECL, Thermo Fisher Scientific, Villebon-sur-Yvette, France) on the ChemiDoc XRS+ apparatus (Bio-Rad Laboratories).

### 2.7. PCR Stop Assay

Cells were seeded at a density of 20,000 cells·cm^−2^ and allowed to adhere for 24 h before treatment with the indicated compounds. DNA was extracted using a genomic DNA isolation kit (Qiagen; Courtabœuf, France). Cisplatin or ifosfamide adduct formation was analyzed by a PCR-based DNA damage assay as previously described [17], using forward, reverse, and nested primer sequences for murine samples was followed: 5′-TGCCGAGGATTTGGAAAAAGT-3′, 5′-ATACTTACACATAGCTCTT-CAGTC-3′, and 5′-CCATCACATTGTGGCCCTCT-3′, respectively. PCR products were resolved on 1.2% and 0.8% agarose gels, and signal quantified using ImageJ software package (v1.53c; National Institutes of Health, Bethesda, MD, USA).

### 2.8. Automated Image Acquisition and Analysis

Cells were seeded onto glass slides at a density of 12,000 cells·cm^−2^ and allowed to adhere for 24 h before treatment with the indicated compounds. Cells were then washed twice in PBS, fixed for 1 h at +4 °C in 1% paraformaldehyde, and imaged out on a fluorescence microscope (Vectra 3 Perkin Elmer) equipped with a digital camera. Nuance multispectral image cubes (8-bit) were acquired with a 20× objective lens (0.5 micron/pixel) and using a full CCD frame at 1 × 1 binning (1360 × 1024 pixels). Fluorescence intensity reflecting the concentration-dependent accumulation of doxorubicin in cultured cells was detected using spectral characteristics corresponding to Cy3. Ten random fields were selected for each condition and quantified using the ImageJ software package.

### 2.9. Trapped in Agarose DNA Immunostaining (TARDIS) Assay

Topoisomerase II binding to DNA was evaluated as previously described [18,19]. Briefly, cells were embedded in low melting-point agarose (1% in PBS) and spread onto glass slides. Cells were incubated for 15 min in lysis buffer (1% SDS, 80 mM PBS, 10 mM ethylene diamine tetra acetic acid (EDTA), 1 mM 1,4-dithiothreitol (DTT); 1X cocktail of protease inhibitor) then for 30 min in 1 M NaCl. After three washes in PBS, slides were incubated for 1 h at RT in a humidified chamber with a rabbit anti-TOP2B (#4555) antibody (1/50 in 1% BSA, 0.1% Tween-20 in PBS), washed three times in PBS/0.1% Tween-20, then incubated for 1 h with Alexa-647-coupled anti-rabbit secondary antibody (1/500 in 1% BSA, 0.1% Tween-20 in PBS). Slides were finally counterstained for 1 min with 4′,6-diamidino-2-phenylindole (DAPI) and examined by an inverted epifluorescence microscope set at 20X magnification (EVOS-FL; Thermo Fisher Scientific). For each sample, sixty to one hundred nuclei were examined in randomly chosen fields, and signal intensity was determined in Topo II-positive cells using the ImageJ software package.

### 2.10. Immunofluorescence

Cells were seeded at a density of 12,000 cells·cm^−2^ and allowed to adhere for 24 h before treatment with the indicated compounds. Cells were then washed twice in PBS, fixed for 1 h at +4 °C in 1% paraformaldehyde, and permeabilized for 5 min at RT with 0.1% Triton-X100. After the saturation of potential nonspecific binding sites for 1 h with 1% BSA, cell layers were incubated for 1 h at RT with a rabbit anti-β-Tubulin (ab6046; Abcam) antibody (1/1000 in 1% BSA), washed three times in PBS/0.1% Tween-20, then incubated for 1 h at RT with Alexa-647-coupled anti-rabbit secondary antibody (1/1000 in 1% BSA). Wells were finally counterstained for 1 min with DAPI and examined by a virtual slide microscope (Olympus VS120) at 20× magnification. Fluorescence signal intensity was evaluated using the ImageJ software package.

### 2.11. In Vitro Wound-Healing Assay

Wounding assay was performed according to the manufacturer’s instructions (Ibidi, Martinsried, Germany). Confluent cell monolayers were cultured for 18 h, fixed in 75% ethanol, then stained with crystal violet (0.05% in ethanol). Recovery of the denuded area was computerized using an inverted digital microscope (EVOS) and evaluated using ImageJ software package. Lesion area surface at time zero was used as a matrix.

### 2.12. Cell Invasion Assays

In vitro cell invasion was evaluated as previously described [10] using transwell Boyden chambers (8 µm pore size; BD Biosciences, San Jose, CA, USA) coated with basement membrane Matrigel. Two independent assays were performed in triplicates for each condition. Invading cells were counted onto eight fields randomly selected on each membrane.

### 2.13. Matrix Metalloproteinase-2 Activity Assay

Matrix metalloproteinase-2 (MMP2) activity was evaluated by a colorimetric assay as previously described [10]. The activity was expressed as treated over control ratio after correction for total protein content.

### 2.14. Cell-Derived Xenograft (CDX) Models

All animal experiments and procedures were approved by the local French ethical animal committee of Paris Saclay University (Comité d’Ethique pour l’Expérimentation Animale CEEA 26) and conducted in a pathogen-free environment. After acclimatization for 7 days, 6-week-old BALB/c homozygous athymic nude mice (nu/nu; Charles River, Arbresle, France) were injected in both thigh muscles with 10^6^ cells in 15 µL PBS under isoflurane/air anesthesia. After randomization at day 11 post tumor cells injection, mice were treated with doxorubicin (4 mg·kg^−1^; intraperitoneally once a week), cisplatin (7 mg·kg^−1^; intraperitoneally once a week), simvastatin (15 mg·kg^−1^ per day in drinking water), combination doxorubicin + simvastatin, combination cisplatin + simvastatin, or PBS. At 1 month post tumor cells injection, mice were killed by cervical dislocation. Based on caliper measurements, the tumor volume was calculated using the formula: (π/6) × W × L × D where W is the tumor width, L is the tumor length, and D is the tumor depth. Thigh muscles infiltrated with tumor tissues and lungs were fixed in 4% paraformaldehyde in PBS, then paraffin-embedded.

### 2.15. Immunohistochemistry

Formalin-fixed paraffin-embedded (FFPE) tissue sections (4 µm) were deparaffinized in xylene and rehydrated through a graded series of ethanol before hematoxylin and eosin staining (H&E). In situ cell death detection was performed by terminal deoxynucleotidyl transferase-mediated dUTP nick-end labeling (TUNEL) assay according to the manufacturer’s recommendation (Roche Diagnostics, Boulogne-Billancourt, France). Slides were revealed with permanent red (Dako-Agilent Technologies, Courtabœuf, France) and counterstained with hematoxylin. Each slide was digitalized using an Olympus VS120 slide scanner. Quantitation of TUNEL-positive cells was performed as previously described [20], using the ImageJ software package.

### 2.16. Statistical Procedures

Statistical analyses were performed using Graph Pad Prism 7 software. A one-way ANOVA (Dunnet’s multiple comparison test) was used to compare cell viability under treatments. A one-way ANOVA (Mann–Whitney test) was used to compare the doxorubicin fluorescence content, TARDIS. A one-way ANOVA (Tukey multiple comparison test) was used to compare signal intensity in TARDIS assay, to compare tubulin immunofluorescence signal intensity, and to compare MMPs activities. A two-way ANOVA (Tuckey multiple comparison test) was used to compare in vivo criteria. Significance was set at a *p*-value of 0.05.

## 3. Results

### 3.1. Simvastatin Alters Cell Membrane Permeability

**Figure 1:**
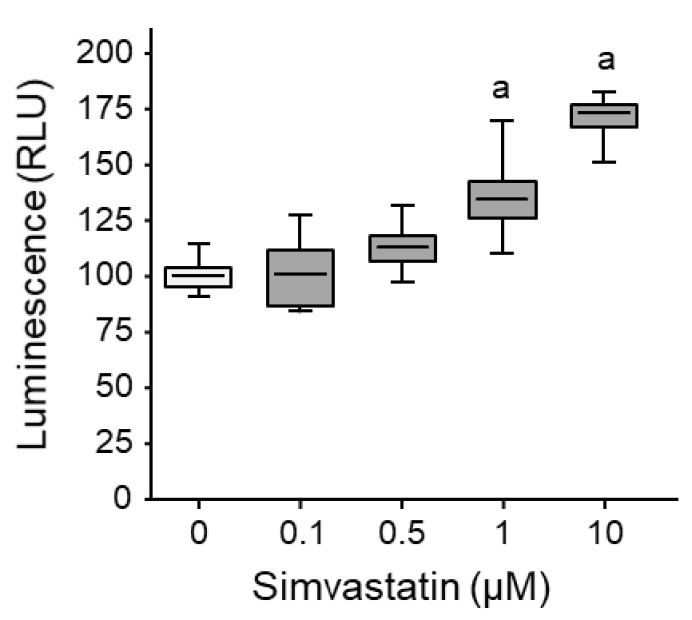
Statin-induced osteosarcoma cell membrane permeability. Murine osteosarcoma cells K7M2 were loaded with calcein-AM (5 %M) for 2 h, then exposed to increasing concentrations of simvastatin for 16 h. Calcein release during plasmolysis induced by a hyperosmotic solution was determined. Box plot illustrating the global relative luminescence units (RLU). Lower, middle, and top lines of boxes indicate lower quartile, median, and upper quartile, respectively. Whiskers indicate minima and maxima. a: *p* < 0.002 compared to solvent.

We evaluated the plasma membrane fluidity and permeability of K7M2 murine osteosarcoma cells to increasing doses of simvastatin. The fluorescence signal intensity dose-dependently increased (up to +71% at 10 µM; Figure 1).

These results suggest that simvastatin alters murine osteosarcoma cell membrane fluidity and permeability.

### 3.2. Simvastatin and Chemotherapy Synergistically Reduce Cell Viability In Vitro

**Figure 2:**
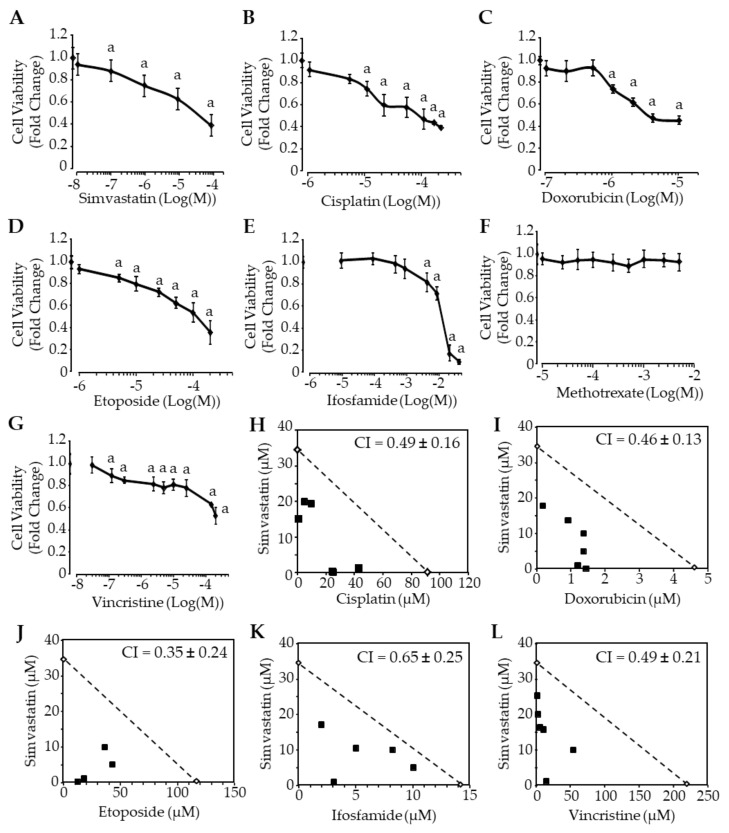
Chemotherapy drugs and simvastatin synergistically reduce osteosarcoma cell viability in vitro. (**A**–**G**) Concentration-dependent cytotoxicity of conventional chemotherapy drugs and simvastatin. Murine osteosarcoma cells K7M2 were exposed to increasing concentrations of the indicated compounds for 24 h. Cell viability was evaluated by the MTT test. Results are expressed as mean ± SD (*n* = 6–8) of four independent experiments. a: *p* < 0.05 compared to solvent. (**H**–**L**) Isobologram analyses of agent combination. Cells were exposed to agent alone (IC_50_) or in combination (at various fixed concentration ratios) for 24 h. Cell viability was evaluated by the MTT test to determine the IC_50_ values of the different combinations. The isobole (dotted) line represents the expected additive effect when there is no interaction between the drugs. The combination index (CI) was determined as described in the Materials and Methods section.

We determined the sensitivity of K7M2 cells to increasing doses of simvastatin or chemotherapy (cisplatin, doxorubicin, etoposide, ifosfamide, methotrexate, and vincristine). All tested drugs dose-dependently reduced cell viability (Figure 2), except methotrexate that did not exhibit significant cytotoxicity up to concentrations as high as 5 mM (Figure 2F). The half-maximal inhibitory concentrations (IC_50_) are reported in Table 2.

We next investigated the effect of combined therapy. Cells were treated for 24 h with dose-escalating of single and combined drugs before cell viability assessment by the MTT test. An isobologram analysis was performed to evaluate synergism, additive effect, or antagonism between simvastatin and chemotherapy. Calculation of combination index (CI) revealed values lower than 1 for all tested combinations, indicating a synergistic effect of simvastatin with chemotherapy (Figure 2H–L).

Taken together, these results suggest that simvastatin and chemotherapy reduce murine osteosarcoma cell viability in vitro and can exert a synergistic effect when combined.

### 3.3. Simvastatin Prevents RhoA GTPase Geranylgeranylation

Statins competitively block the active site of the HMGCR, the first and key rate-limiting enzyme in the endogenous mevalonate pathway [21]. We investigated the resulting effect of mevalonate pathway blockade by simvastatin on K7M2 cell viability. Cells were pretreated for 1 h with increasing concentrations of mevalonate, geranylgeranyl pyrophosphate (GGPP), or farnesyl pyrophosphate (FPP) before exposure to simvastatin for a further 24 h. Mevalonate or GGPP supplementation fully prevented the inhibitory effect of simvastatin on cell viability (Figure S1A,B), whereas pretreatment with FPP failed to interfere with the simvastatin inhibitory effect (Figure S1C).

FPP and GGPP are substrates for protein prenylation, an essential post-translational modification consisting in the covalent anchorage of such lipid residues onto specific proteins such as GTPases that allows their translocation from the cytosol to the membrane [22]. We evaluated RhoA protein content in the cytosolic fraction of cell lysate. Treatment with simvastatin resulted in RhoA relocation from the membrane to the cytosol (Figure S1D), confirming that inhibition of HMGCR activity by simvastatin prevents RhoA prenylation. Pretreatment with GGPP prevented the RhoA relocation to the cytosol induced by simvastatin, whereas pretreatment with FPP had no effect (Figure S1D).

Taken together, these results suggest that simvastatin alters RhoA protein prenylation through depletion of the mevalonate intermediate geranylgeranyl pyrophosphate (GGPP) in our murine osteosarcoma cells.

### 3.4. Simvastatin Favors Cisplatin and Ifosfamide Access to DNA

**Figure 3:**
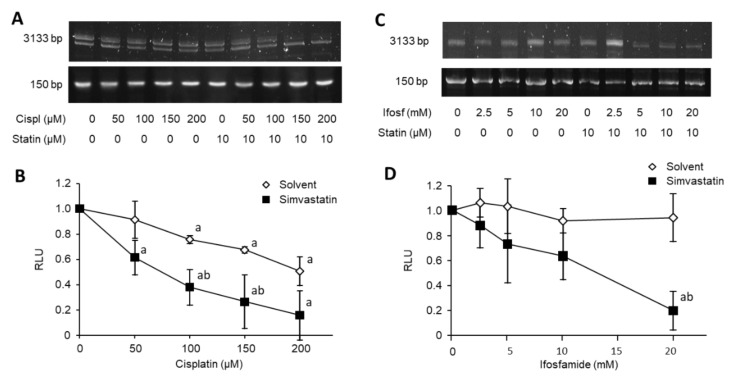
Simvastatin favors the formation of DNA adducts with cisplatin and ifosfamide. (**A**–**C**) Analysis of PCR products by agarose gel electrophoresis. Cells were exposed to increasing concentrations of cisplatin (A) or ifosfamide (**C**) for 1 h in the presence of simvastatin (10 µM) or solvent. Genomic DNA was extracted and subjected to a PCR stop assay. (**B**–**D**) PCR amplification efficacy according to the indicated drug concentration for solvent- or simvastatin-treated cells. Results are expressed as mean ± SD (*n* = 3) of relative quantification of a signal after normalization for the nested fragment. a: *p* < 0.05 vs. untreated, b: *p* < 0.05 vs. solvent.

We investigated DNA damage following exposure to increasing concentrations of cisplatin and ifosfamide, alone or in combination with simvastatin. The DNA-drug adducts accumulation was assessed using PCR stop assay. As expected, exposure for 30 min to high concentrations of cisplatin inhibited DNA amplification (from −24% at 100 µM to −49% at 200 µM; Figure 3A,B). The combination of simvastatin with cisplatin led to greater inhibition of PCR amplification than cisplatin alone (from −40% at 50 µM to −84% at 200 µM). Similarly, exposure for 30 min to ifosfamide slightly inhibited DNA amplification (−10% at >10 mM; Figure 3C,D). The combination of simvastatin with ifosfamide led to greater DNA damage than ifosfamide alone (up to −80% at 20 mM).

### 3.5. Simvastatin Favors Doxorubicin Accumulation into Nucleus

**Figure 4:**
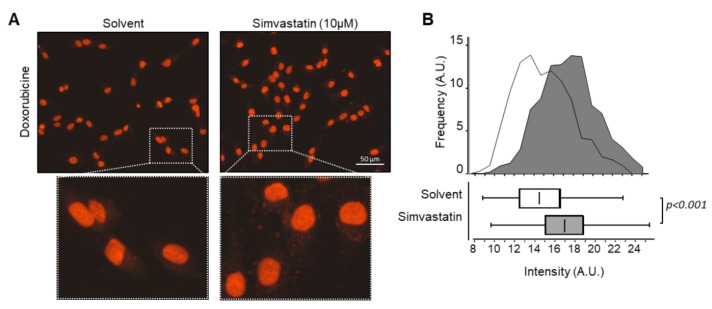
Simvastatin favors doxorubicin accumulation into osteosarcoma cells nuclei. (**A**) Intracellular uptake of doxorubicin. Cells were exposed to doxorubicin (3.6 µM) for 30 min in the presence or absence of simvastatin (10 µM). The image was taken by fluorescent microscope equipped with a digital camera. Enlarged images are shown in the inserts. (**B**) Distribution of red fluorescent signal intensity as a function of the frequency of occurrence for vehicle-treated cells (light gray) and simvastatin-treated cells (dark gray). Results are also expressed as a box plot. Lower, middle, and top lines of boxes indicate lower quartile, median, and upper quartile, respectively. Whiskers indicate minima and maxima.

We investigated the subcellular accumulation of doxorubicin following 30 min of exposure to the drug alone or in combination with simvastatin. Doxorubicin localized mainly to the nuclei (Figure 4A). The red fluorescence intensity in the nuclei was 1.4-fold higher for cells treated with the combination of doxorubicin and simvastatin than for cells treated with doxorubicin alone (Figure 4B).

These results suggest that simvastatin favors doxorubicin accumulation in the nucleus of our murine osteosarcoma cells, which may favor DNA damage through intercalation or direct alkylation. These results are consistent with the synergistic effect on cell viability reduction.

### 3.6. Simvastatin Favors Etoposide Access to DNA

**Figure 5:**
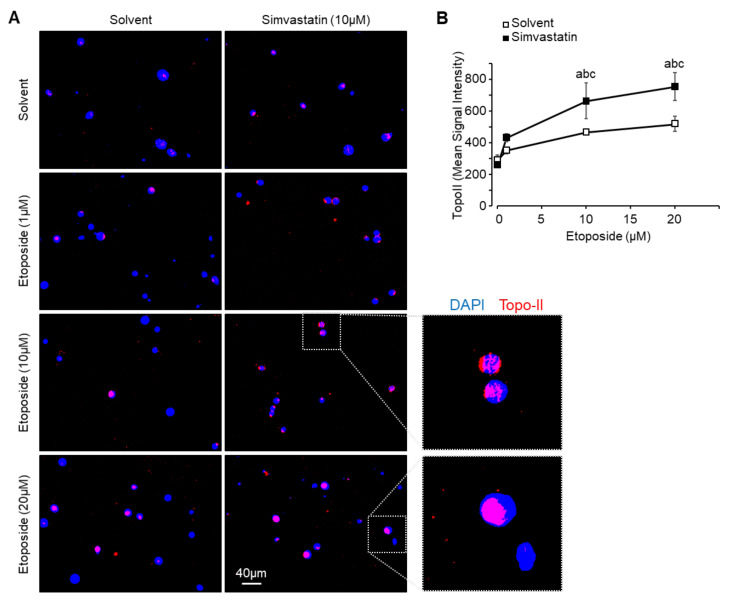
Simvastatin favors etoposide access to DNA. (**A**) Concentration-dependent topoisomerase II (Topo II)-DNA adducts formation. Cells were exposed to increasing concentrations of etoposide for 1 h in the presence or absence of simvastatin (10 µM) and subjected to TARDIS analysis. Immunofluorescence staining for merged DAPI stained nuclei (blue) and Topo II signal detected using Alexa488-labeled secondary antibody (red). (**B**) Quantification of Topo II signal intensity. Results are expressed as mean ± SEM. a: *p* < 0.05 compared to solvent. b: *p* < 0.05 compared to simvastatin alone. c: *p* < 0.05 compared to etoposide alone.

We investigated the topoisomerase II (Topo II)-DNA adducts formation following exposure to increasing concentration of etoposide, alone or in combination with simvastatin. The Topo II-DNA adducts induction was assessed using the TARDIS (trapped in agarose DNA immunostaining) method. As expected, exposure for 1 h to increasing concentration of etoposide dose-dependently stabilized the Topo II-DNA complex (from +20% at 1 µM to 1.8-fold at 20 µM; Figure 5A,B). The combination with simvastatin (30 min pretreatment followed by 1 h co-treatment) led to enhanced fluorescence signal intensity compared to etoposide alone for all tested concentrations (+65% at 1 µM to 2.9-fold at 20 µM).

These results suggest that simvastatin favors the etoposide-dependent inhibition of Topo II activity in our murine osteosarcoma cells. These results are consistent with the synergistic effect on cell viability reduction.

### 3.7. Simvastatin Favors Vincristine Destabilization of Microtubules

**Figure 6:**
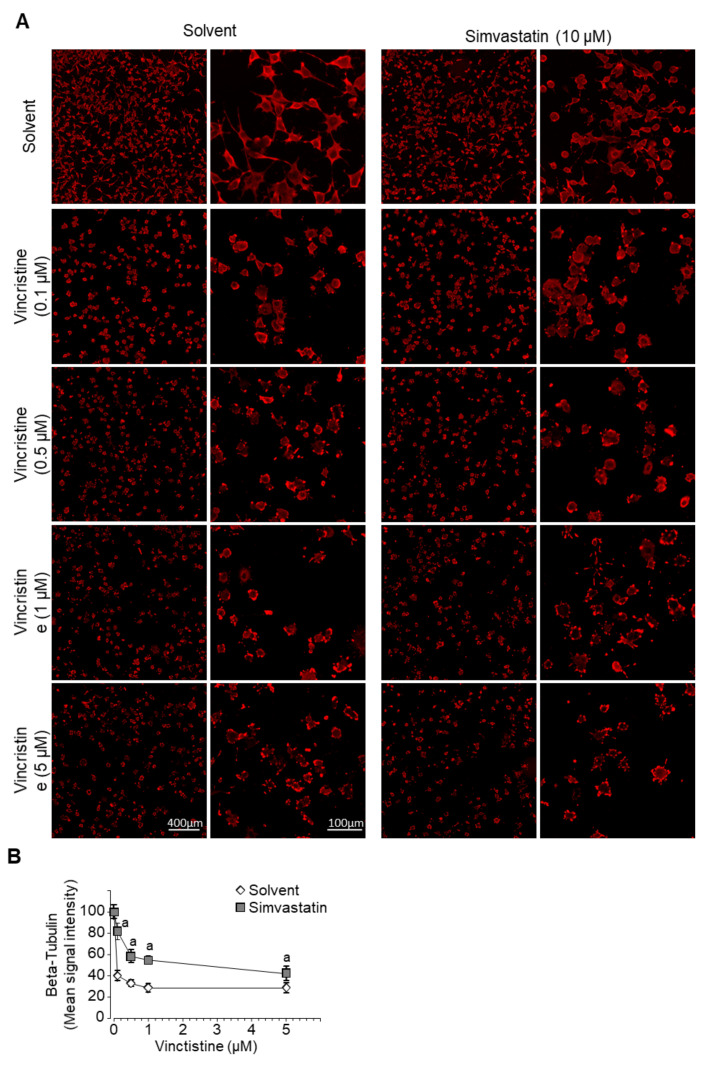
Simvastatin favors microtubules destabilization by vincristine. (**A**) Microtubule structure visualized by fluorescent labeling using an anti-β-tubulin antibody. Cells were exposed to increasing concentrations of vincristine for 6 h in the presence or absence of simvastatin (10 µM). (**B**) Quantification of β-tubulin signal intensity. Results are expressed as mean ± SD (*n* = 8–10). a: *p* < 0.05 compared to solvent.

We investigated microtubule rearrangement following exposure to increasing concentrations of vincristine, alone or in combination with simvastatin. Cells were processed for immunofluorescence staining for β-tubulin. As expected, exposure for 6 h to increasing concentrations of vincristine disrupted microtubule polymerization as reflected by a reduced average signal intensity (from −60% at 0.1 µM to −72% at 5 µM) and altered geometry of β-tubulin signal (Figure 6A,B). The combined treatment with simvastatin moderated the reduction in fluorescence signal intensity (only −18% at 0.1 µM, up to −58% at 5 µM, *p* < 0.05) and amplified the disorganization of the cytoskeleton, as illustrated by the cellular stellate shape.

These results suggest that simvastatin favors the vincristine-dependent disorganization of microtubules in our murine osteosarcoma cells. These results are consistent with the synergistic effect on cell viability reduction.

### 3.8. Simvastatin and Chemotherapy Synergistically Reduce Cell Motility and Invasiveness In Vitro

**Figure 7:**
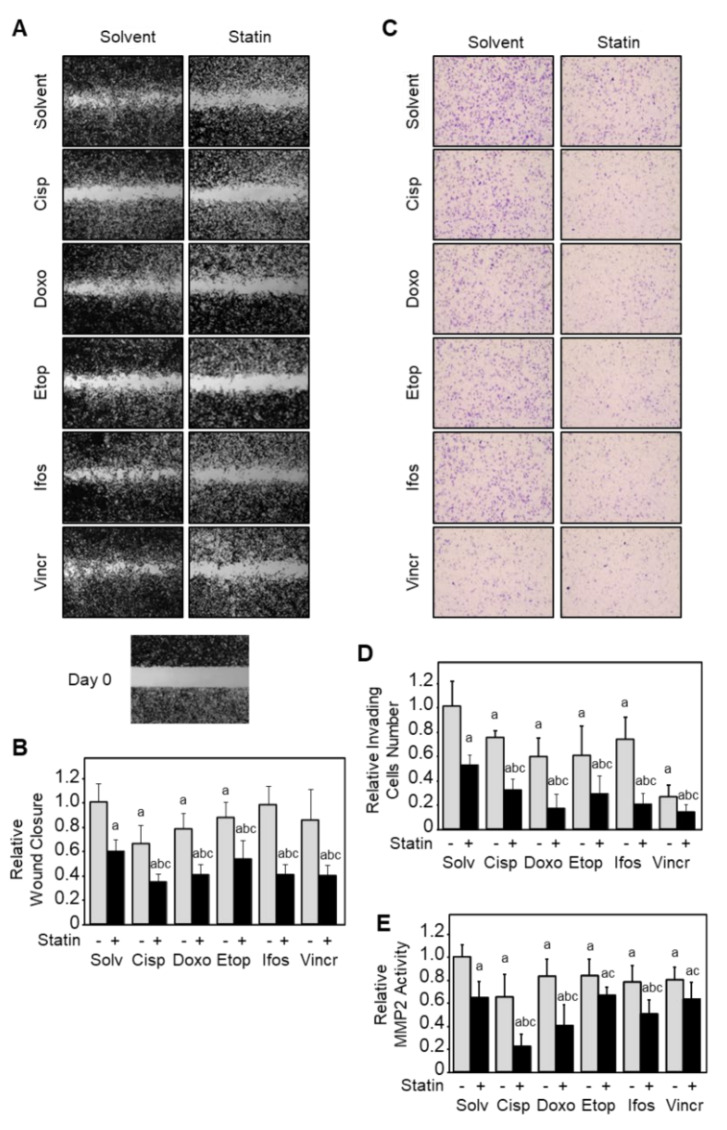
Chemotherapy drugs and simvastatin synergistically reduce osteosarcoma cell invasiveness in vitro. (**A**) Cell motility evaluated by wound-healing assay. Cells were exposed to the single or combined indicated compounds in the presence of the broad caspases inhibitor zVAD-fmk (20 µM). Pictures were taken at times 0 and 18 h after the wound. (**B**) Relative migrating cell number in wound-healing assay. Results are expressed as mean ± SD (*n* = 3–4) of two independent experiments. (**C**) Cell invasion evaluated by Matrigel-coated Boyden chambers. Cells were exposed to the indicated single or combined compounds for 16 h. (**D**) Relative invading cell number. Results are expressed as mean ± SD (*n* = 3) of two independent experiments. (**E**) Matrix metalloproteinase 2 (MMP2) activity evaluated by a colorimetric assay. Cells were exposed to the single or combined indicated compounds for 24 h. Results are expressed as mean ± SD (*n* = 5–6) of two independent experiments. a: *p* < 0.05 compared to untreated, b: *p* < 0.05 compared to simvastatin alone, c: *p* < 0.05 compared to chemotherapy drug alone.

We evaluated the in vitro cell motility and cell invasiveness following exposure to chemotherapy drugs alone or in combination with simvastatin. The cell motility was assessed by a wound-healing assay, and cell invasiveness was assessed using Matrigel-coated modified Boyden chambers. To prevent a possible bias due to the reduction in cell viability, all experiments were performed in the presence of zVAD-fmk (20 µM), a broad-spectrum caspases inhibitor. As expected, chemotherapy and simvastatin alone reduced migrating cells number (up to −34% vs. solvent; Figure 7A,B). The combination of simvastatin with chemotherapy led to a stronger inhibitory effect on migrating cell numbers than each chemotherapy alone (+38% to +58% vs. drug alone).

We next evaluated the potential of K7M2 cells to invade the extracellular matrix. Cells were pre-incubated with the indicated compounds for 2 h before seeding into Boyden chambers coated with Matrigel and incubated for another 22 h. As expected, chemotherapy or simvastatin reduced invading cell number (up to −74% vs. solvent; Figure 7C,D). The combination of simvastatin with any chemotherapy drugs led to a stronger inhibitory effect than chemotherapy alone (+45% to +70% vs. drug alone).

We finally evaluated the matrix metalloproteinase MMP2 activity (Figure 7E). Cells were exposed to the indicated compounds for 24 h, and the enzymatic activity was assessed by a colorimetric assay. Chemotherapy or simvastatin reduced MMP2 activity (up to −38% vs. solvent), and the combination of simvastatin to chemotherapy led to a stronger inhibitory effect than chemotherapy alone (+15% to +63% vs. drug alone).

Taken together, these results suggest that simvastatin reinforces the inhibitory effect of chemotherapy on tumor cells migration and invasion.

### 3.9. Simvastatin Reinforces Osteosarcoma Cells Sensitivity to Chemotherapy In Vivo

**Figure 8:**
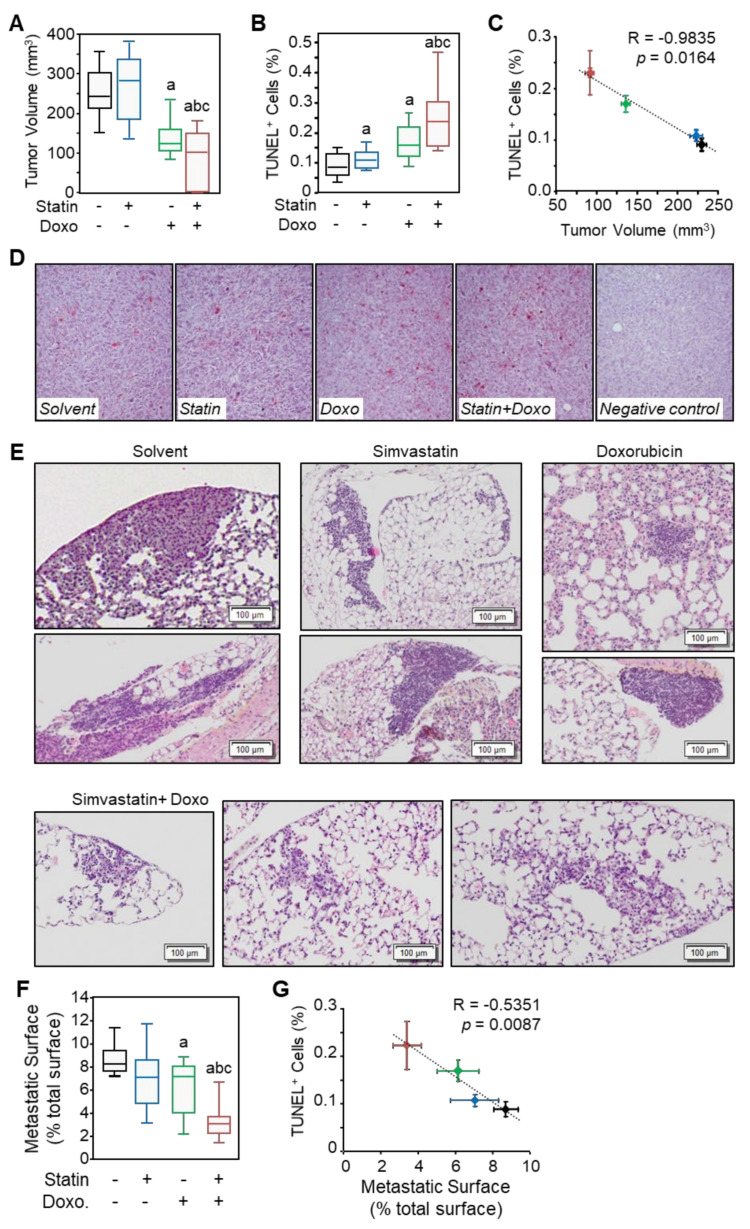
Simvastatin alone does not impair tumor growth and dissemination but enhances the chemotherapy effect. Mice (*n* = 6 per group) were injected intramuscularly with murine osteosarcoma cells K7M2 to develop primary tumors. On day 11, randomized mice received saline (intraperitoneal injection once a week), simvastatin (20 mg kg^−1^ day^−1^ in drinking water), doxorubicin (4 mg kg^−1^; i.p. once a week), or a combination of treatments. (**A**) Tumor volume was determined on day 28, the date of sacrifice. Results are expressed as a box plot. Lower, middle, and top lines of boxes indicate lower quartile, median, and upper quartile, respectively. Whiskers indicate minima and maxima. a: *p* < 0.05 compared to solvent; b: *p* < 0.05 compared to simvastatin alone and c: *p* < 0.05 compared to doxorubicin alone. (**B**–**D**) Tumor cell apoptosis rate was determined by TUNEL assay on FFPE sections. Results are expressed as a box plot. a: *p* < 0.05 compared to untreated cells; b: *p* < 0.05 compared to simvastatin alone and c: *p* < 0.05 compared to doxorubicin alone. (**C**) Correlation between tumor volume and percentage of TUNEL-positive cells. (**E**) Representative sections of the lung stained with H&E show metastatic foci invading the pulmonary tissue. (**F**) Relative metastatic surface was determined to whole lung surface. Results are expressed as mean ± SD (*n* = 6). a: *p* < 0.05 compared to untreated cells; b: *p* < 0.05 compared to simvastatin alone and c: *p* < 0.05 compared to doxorubicin alone. (**G**) Correlation between lung metastatic surface and percentage of TUNEL-positive cells in the primary tumor.

K7M2 cells were inoculated in the thigh muscles of Balb/c nude mice to develop primary tumors. Mono- and combined therapies were started after 11 days when the tumors had reached an average volume of 50–60 mm^3^. Unfortunately, cisplatin administration induced various toxicities leading to loss of weight and ethical discontinuance of experimental procedures. For the other groups, at sacrifice, tumors were measured before formalin fixation and paraffin embedding. As expected, doxorubicin administration reduced tumor volume (−40% vs. control; Figure 8A). Simvastatin alone did not modify tumor growth, but the combination with doxorubicin led to a stronger inhibitory effect than doxorubicin alone (−60% vs. control).

Four-micron sections of FFPE tissues were processed for detecting fragmented DNA in apoptotic cells by TUNEL assay (Figure 8B,D). As expected, doxorubicin administration increased TUNEL-positive cell number in tumor samples (1.8-fold vs. control). Simvastatin alone did not affect TUNEL-positive cell number, but combination to doxorubicin led to a stronger pro-apoptotic effect than doxorubicin alone (2.7-fold vs. control). Tumor volume and the number of TUNEL-positive cells were negatively correlated (Figure 8C).

Taken together, these results suggest that simvastatin reinforces the inhibitory effect of chemotherapy on tumor cell growth in a preclinical model.

### 3.10. Simvastatin Reinforces Chemotherapy Effect on Osteosarcoma Cells Dissemination In Vivo

Intramuscular generated tumors disseminated and led to the occurrence of metastatic foci. At the time of mice sacrifice, lungs were harvested, fixed with formalin, and embedded in paraffin. Metastases presence and area were evaluated on H&E stained sections (Figure 8E). As expected, doxorubicin administration reduced metastatic tissue surface (−30% vs. control; Figure 8F). Simvastatin alone did not modify metastatic tissue surface, but combination to doxorubicin led to a stronger inhibitory effect than doxorubicin alone (−60% vs. control). The metastatic tissue surface and the number of TUNEL-positive cells were negatively correlated (Figure 8G).

Taken together, these results suggest that simvastatin reinforces the inhibitory effect of chemotherapy on tumor cell metastatic dissemination in a preclinical model.

## 4. Discussion

Chemoresistance is a critical issue for osteosarcoma. Patients who fail or progress on neoadjuvant and post-operative multi-drug chemotherapy protocols are of poor prognosis. The 5-year overall survival is stagnating at about 60% since the late 1970s despite considerable but disappointing efforts to identify and test more efficient treatments such as targeted agents [1]. In the present study, we report the synergistic effect of simvastatin in combination with conventional chemotherapy drugs in osteosarcoma. Our results illustrate that simvastatin enhances anti-tumor activities of chemotherapy in vitro and in a preclinical model. This suggests that such a combination of drugs may be of significant therapeutic interest in osteosarcoma.

Numerous studies showed that the mevalonate pathway is up-regulated in hematologic and solid tumors [23]. Because nitrogen-containing bisphosphonates inhibit the mevalonate pathway, they are, for example, proposed in veterinary practice for the palliative care of bone tumors [24]. Targeting this metabolic pathway, especially with zoledronic acid, has been widely investigated in the context of osteosarcoma [25,26,27,28,29]. Based on numerous promising in vitro and preclinical results of coupling bisphosphonate to conventional chemotherapy [27,30,31,32,33], and despite some negative assays [34,35], several randomized phase III clinical trials were launched [36,37]. However, none highlighted any improvement in clinical outcomes.

We focused our work on statins that also affect the mevalonate pathway but upstream of bisphosphonates by inhibiting the 3-hydroxy-3-methylglutaryl-coenzyme A reductase (HMGCR) activity. Statins are historically used as a cholesterol- and lipid-lowering medications. However, their therapeutic interest in cancer revealed in in vitro and preclinical models [6] and sustained by epidemiological and next-generation sequencing data [22,38] has been confirmed by benefits in numerous clinical trials [7]. Statins also showed beneficial effects as combined therapy with chemotherapy. As an example, statins have a favorable effect on the response to gemcitabine and erlotinib in advanced pancreatic cancer [39]; or the response to thalidomide, carboplatin/vincristine in pediatric brain tumors [7,40]. Statins also prolong the survival of patients with advanced chemo-resistant hepatocellular carcinoma [41] or metastatic renal cell carcinoma treated with various targeted therapies [42]. With regard to osteosarcoma, no epidemiological data are available on possible anti-tumor effects of statins in this childhood cancer. Only one in vivo study investigated statins as combination therapy in a preclinical murine model of osteosarcoma and reported a synergistic interaction with another hypocholesterolemic agent targeting HMGCR (apomine), resulting in an enhanced anti-tumor effect [12]. To our knowledge, no data are available to estimate the interest of statins as adjuvant therapy in osteosarcoma, even for patients that are poor responders to chemotherapy.

We previously demonstrated that lipophilic statins (atorvastatin, simvastatin, or cerivastatin) trigger caspases-dependent apoptosis in osteosarcoma cells [43] and reduce cell invasiveness through inhibition of a RhoA GTPase-JNK-MMP2 cascade [10]. We also demonstrated in vitro that statins sensitize human osteosarcoma cells to chemotherapeutic drugs, resulting in reduced cell viability, migration, and invasion [11]. Taken together, these results show that cholesterol-lowering agents exhibit a promising ability to increase the responsiveness of osteosarcoma cells to classical anticancer cytotoxic drugs. Further investigations are required to characterize the mechanism of synergism. Given the multiplicity of the target for drugs (DNA strand, topo-isomerase, microtubules…), it is unlikely to identify a common molecular/protein target for statins. We detected a modulation of the membrane properties presumably based on the hypocholesterolemiant purpose of statins. The cholesterol content of the bilayer is indeed one of the main factors that influence membrane fluidity and permeability. Cholesterol helps to restrict the passage of molecules by increasing the packing of phospholipids. Therefore, by decreasing the cholesterol content, simvastatin may contribute to increasing the membrane’s fluidity (as described by [44]) and favor the active and/or passive diffusion of drugs. In accordance, we reported here some evidence of simvastatin-dependent drug accumulation and enhanced anti-tumor effects of tested drugs. Simvastatin combination would help to overcome some of the mechanisms of resistance involving variation in drug efflux or through the activation of cell surface receptors (reviewed in [45]).

The present study evaluates the effect of simvastatin in combination with chemotherapeutic drugs in a murine model. Our results support previous studies on human cell lines [10,11,43], confirming additive and/or synergistic anti-tumor effects of statins when combined with chemotherapy. Furthermore, we showed for the first time in a preclinical osteosarcoma model that hypocholesterolemic agents reinforce chemotherapy cytotoxicity in a synergistic manner. We also observed that in contrast to doxorubicin, which exerts anti-tumor effects on its own, simvastatin alone has no effects on cell survival. This is consistent with studies investigating statins as monotherapy in advanced-stage cancer that have not led to significant results [7]. The major limitation of this study is that the cisplatin regimen generated rapid weight loss despite intraperitoneal pre-hydration with an injection of saline solution. This high toxicity in mice triggered the ethical discontinuance of the experimental procedure. Tumors derived from mice treated with cisplatin alone or combined with simvastatin were not analyzed. However, isobologram analyses provide evidence of a synergistic effect of the association of cisplatin to simvastatin on in vitro cell viability. The combination index value (0.49) was comparable to that derived from the association of doxorubicin and simvastatin (0.46), both clearly indicating a synergistic effect. Despite the absence of mouse data, we can hypothesize that a combination of cisplatin and simvastatin can also result in synergistic or at least additive effects *in vivo*.

An important point is to estimate whether a therapeutically efficient concentration of statins derived from in vitro assays can be achieved in vivo. Based on the method described by Xu et al. [46], the plasma concentration of simvastatin in our in vivo experiments may be estimated at around 10–50 nM, which is close to but under the lower efficient concentration, we detected in vitro. This could explain the absence of perceptible effect of simvastatin as monotherapy *in vivo* while still having a beneficial effect as adjuvant therapy.

The administration of statins as therapy is topical as a first-line pharmacologic intervention for pediatric patients with severe dyslipidemias and/or at high risk of cardiovascular disease since the last few years [47,48,49]. In the adult population, the serious adverse events that could be associated with long-term statin therapy are myalgia, myopathy (muscle weakness associated with variations in serum concentrations of creatine kinase), and new-onset type 2 diabetes [50]. All of them can be settled after stopping treatment or minimized by ubiquinone supplementation.

## 5. Conclusions

The aim of the present study was to assess the potential benefit of statin as an adjuvant to chemotherapy in osteosarcoma. We show that simvastatin synergizes with conventional chemotherapy drugs in terms of cell viability, tumor growth, and dissemination. Altogether, our results suggest that simvastatin may represent a valuable alternative adjuvant therapy to potentiate chemotherapy anti-tumor activity in osteosarcoma tumors. Due to their synergistic activity, downsizing chemotherapy dosage might be considered to reduce pernicious side effects. The highlighted synergistic effect of simvastatin combination with commonly used chemotherapy drugs in comparison to monotherapy observed in a preclinical model needs further investigation in clinical trials.

## Figures and Tables

**Table 1:** Details of stock solutions.

Compound	CAS Number	Solvent	Concentration
Cisplatin	15663-27-1	H_2_O	50 µM
Doxorubicin	23214-92-8	H_2_O	18.4 mM
Ifosfamide	3778-73-2	H_2_O	250 mM
Vincristine	57-22-7	H_2_O	1.21 mM
Simvastatin	79902-63-9	DMSO	10 µM
Farnesyl pyrophosphate	13058-04-3	DMEM	10 mM
Geranylgeranyl pyrophosphate	6699-20-3	DMEM	1 mM
zVAD-fmk	187389-52-2	DMSO	20 mM

**Table 2:** IC_50_ values of drugs in the murine K7M2 cell line, as assessed by the MTT test after 24 h of exposure. *n.d.* not determined.

Compound	IC50 (µM)
Simvastatin	34.57
Cisplatin	99.14
Doxorubicin	3.59
Etoposide	120.5
Ifosfamide	14.26
Methotrexate	*n.d.*
Vincristine	218.8

## Data Availability

No new data sets were created or analyzed in this study. Data sharing is not applicable to this article.

## Supplementary Materials

The following are available online at https://www.mdpi.com/article/10.3390/cancers13225869/s1, Figure S1: Simvastatin-reduced osteosarcoma cell viability involves GGPP-dependent prenylation.
