# Supplementary material for: Synergistic Anti-Tumor Effect of Simvastatin Combined to Chemotherapy in Osteosarcoma
Source: Cancers (Basel). 2021 Nov 22;13(22):5869. doi: 10.3390/cancers13225869 (PMC8616540; doi:10.3390/cancers13225869)
Supplement: Supplementary file 1 [file cancers-13-05869-s001.zip › cancers-1449470-supplementary.pdf]

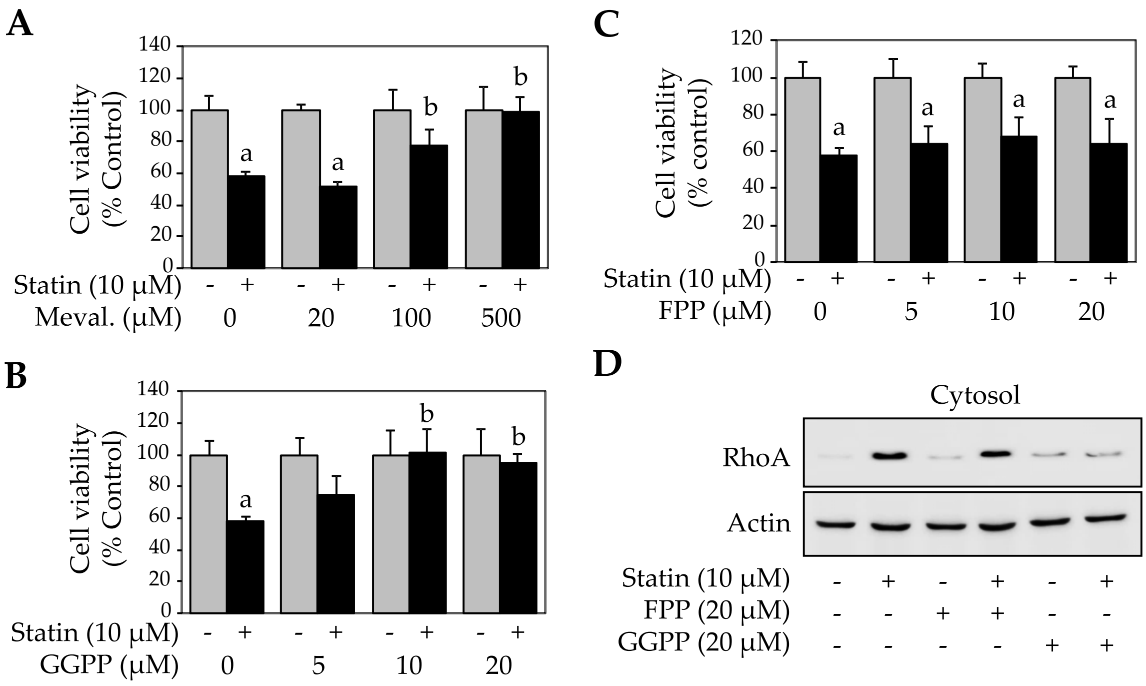

Supplemental Figure S1. Statin-reduced Osteosarcoma Cell viability Involves GGPP-dependent prenylation. (A-C) Murine osteosarcoma cells K7M2 were pretreated for 1 h with increasing concentrations of (A) mevalonate, (B) GGPP or (C) FPP before treatment with/without simvastatin for 24 h. Cell viability was evaluated by the MTT test. Results are expressed as mean ± SD (n=6-8) of four independent experiments. a: $P < 0.05$ compared to pretreatment alone.; b: $P < 0.05$ compared to simvastatin alone. (D) Cells were pretreated for 1 h with FPP (20 µM) or GGPP (20 µM) before treatment with/without simvastatin (10 µM) for 24 h, then lysed and submitted to subcellular fractionation. The RhoA-GTPase expression level in the cytosolic fractions was determined by western blot analysis. Beta-actin was used as loading control.
